# Rapid Multiplexed Immunoassay for Detection of Antibodies to Kaposi’s Sarcoma-Associated Herpesvirus

**DOI:** 10.1371/journal.pone.0163616

**Published:** 2016-09-26

**Authors:** Cathy Logan, Kathryn Todorof, Suzanne P. Fiorillo, Thomas B. Campbell, John H. Elder, Margaret Borok, Ivy Gudza, Lovemore Gwanzura, Buxton Ndemera, Michael J. Lochhead, Constance A. Benson, Robert T. Schooley

**Affiliations:** 1 Department of Medicine, Division of Infectious Diseases, University of California San Diego, San Diego, CA, United States of America; 2 MBio Diagnostics, Inc., Boulder, CO, United States of America; 3 Department of Medicine, Division of Infectious Diseases, University of Colorado Denver, Aurora, CO, United States of America; 4 Department of Immunology and Microbial Science, The Scripps Research Institute, La Jolla, CA, United States of America; 5 Department of Medicine, University of Zimbabwe College of Health Sciences, Harare, Zimbabwe; 6 Departments of Medicine and Medical and Laboratory Sciences, University of Zimbabwe College of Health Sciences, Harare, Zimbabwe; Institut Pasteur of Shanghai Chinese Academy of Sciences, CHINA

## Abstract

Diagnosis of KSHV-infected individuals remains a challenge. KSHV prevalence is high in several populations with high prevalence of HIV, leading to increased risk of development of Kaposi’s sarcoma (KS). While current assays are reliable for detecting antibodies to KSHV, none are routinely utilized to identify individuals with KSHV infection and thus at increased risk for KS due to assay complexity, lack of access to testing, and cost, particularly in resource-limited settings. Here we describe the addition of KSHV proteins LANA and K8.1 to a previously evaluated HIV/co-infection multiplexed fluorescence immunoassay system. This study demonstrates assay performance by measuring antibody reactivity for KSHV and HIV-1 in a collection of clinical specimens from patients with biopsy-proven KS and sourced negative controls. The KSHV assay correctly identified 155 of 164 plasma samples from patients with biopsy-proven KS and 85 of 93 KSHV antibody (Ab)-negative samples for a sensitivity of 95.1% and specificity of 91.4%. Assay performance for HIV-1 detection was also assessed with 100% agreement with independently verified HIV-1 Ab-positive and Ab-negative samples. These results demonstrate good sensitivity and specificity for detection of antibody to KSHV antigens, and demonstrate the potential for multiplexed co-infection testing in resource-limited settings to identify those at increased risk for HIV-1-related complications.

## Introduction

Kaposi’s sarcoma-associated herpesvirus (KSHV), also referred to as human herpesvirus 8 (HHV-8), is a γ-herpesvirus related to the Epstein-Barr virus[1–3], and is the etiologic agent associated with Kaposi’s Sarcoma (KS), Multicentric Castlemans’s Disease (MCD), and Primary Effusion Lymphoma (PEL)[1–7]. KS is a common malignancy among human immunodeficiency virus type 1 (HIV-1)-infected persons, however it can also occur in non-HIV infected individuals. Other epidemiologic forms include the classical, endemic, and post-transplant forms of disease [8–10]. With effective antiretroviral therapy, the incidence of HIV-related KS has dramatically decreased in developed countries; however incidence remains high in areas where both HIV-1 and KSHV infections are prevalent[8].

The development of reliable, accurate, and inexpensive diagnostic and screening tests for KSHV is critical to assess its prevalence in populations, to screen organ and blood donors, and potentially to determine individual risk of developing KS or other malignancies. The genome of KSHV is 165-kb and encodes for 90 gene products, many of which allow evasion of the host immune system and facilitate persistent infection[9, 11]. Infection with KSHV can be identified by the presence of antibodies to the virus or of nucleic acids in blood or tissue. PCR-based approaches for diagnosis and screening are limited by variable clinical sensitivity[12–21], as well as by complexity and cost. Several studies have shown associations between the level of KSHV DNA and the likelihood of KS disease and the stage of KS disease, thus suggesting it could be used as a prognostic tool. However, many studies show a failure to detect KSHV DNA in patients with known seroreactivity and even with active KS disease. Thus, PCR is less suited for use as a screening tool for KSHV infection, particularly in asymptomatic individuals. Antibody detection via immunofluorescence assays, enzyme-linked immunosorbent assays (ELISAs), and western blotting are available, but these serologic tests are rarely used clinically to identify those who have sub-clinical infection and are thus at risk for development of KS, at least partially due to cost and complexity. An advantage of the antibody detection (serology) assays is that they can potentially be configured in simpler formats than the PCR assays. Serologic tests using recombinant KSHV antigens and multi-antigen algorithms have shown promise[22–26]. Some of the major antigenic proteins identified include the latently expressed v-cyclin and latency associated nuclear antigen (LANA), also known as ORF73, as well as K8.1 and ORF65, which are expressed in the lytic cycle. Recently, excellent sensitivity and specificity were demonstrated using these four antigens in combination, indicating this mixture would be useful in serological screening for KSHV in individuals with or at risk for KS[9, 27].

MBio Diagnostics has developed a rapid diagnostic system for simultaneous and cost-effective testing for HIV and co-infections. This system consists of a simple reader instrument and planar waveguide imaging technology to deliver a panel of serologic immunoassay results using a single drop of blood, serum, or plasma[28]. The MBio system can deliver a serological profile in less than 30 minutes, and therefore could provide the panel of results at the time of HIV screening. After HIV diagnosis, guidelines suggest testing for common co-infections such as hepatitis B and C and syphilis[29]. In settings with KSHV, it would also be beneficial to identify KSHV serostatus at the time of HIV diagnosis to more effectively target antiretroviral therapy and employ other interventions to improve surveillance for KS to aid earlier detection and treatment. The MBio multiplexed serology system can deliver multiple serology results at the time of HIV confirmation, without the overhead costs, complicated laboratory procedures, or complex sample management.

In this study, a simple-to-use, prototype HIV / KSHV antibody detection system is demonstrated on a collection of clinical plasma and serum specimens. Results demonstrate the potential for multiplexed co-infection panel testing in a format with applicability in resource-limited settings.

## Methods

### Clinical samples

A total of 164 human plasma samples were previously obtained from participants enrolled in studies of KS conducted at the University of Zimbabwe College of Health Sciences. Samples were provided under an Institutional Review Board (IRB)-approved protocol at the University of California, San Diego. All samples were obtained from patients with confirmed HIV infection and biopsy-proven KS. A total of 93 human serum controls were sourced through Valley Biomedical (Winchester, VA). Sourced specimens were previously tested by the vendor using FDA-approved methods and found negative for hepatitis B surface antigen, hepatitis C, HIV, and syphilis. These samples are expected to be at low risk for KSHV infection, though KSHV serological status was not performed by the vendor.

### KSHV ELISA

Anti-KSHV antibodies were determined on all samples using a commercially available, purified whole-virus ELISA kit produced by Advanced Biotechnologies, Inc. Sample testing was performed per the general procedure outlined in the package insert. Once the reaction was complete, plates were read by measuring optical density (OD) at the recommended wavelength of 450nm, using a DTC-880 Multimode Plate Reader manufactured by Beckman Coulter. Cutoff values were obtained by averaging the three negative control well readings from the raw data output and multiplying the result by 3.0 per package insert instructions. A result is considered positive (reactive) if the OD/cutoff ratio is ≥1, negative (non-reactive) if OD/cutoff ratio is ≤0.75, and equivocal (borderline) when the OD/cutoff ratio is 0.76–0.99 (per package insert).

### Protein expression and purification

Selection of LANA and K8.1 proteins or fragments thereof was based on published observations for the primary reactivities to these proteins by patient sera[9, 19, 22, 23, 25–27, 30–41]. Amino acid residues corresponding to positions 843–1129 of LANA were chosen for expression, based on predicted likelihood for presence of reactive antibody epitopes. K8.1 clones encompassed the entire protein-coding region for each protein (amino acid sequences shown below). The sequences shown in the GenBank repository for K8.1 (accession No. YP_001129404) and LANA (Accession No. YP_001129431) were used to derive the corresponding DNA regions encoding the protein or protein segment to be employed for protein expression constructs. The DNA sequences were then provided to DNA2.0, Inc. (Newark, CA) for gene synthesis, with codons optimized for expression in *Escherichia coli* (DNA 2.0) with the restriction sites XhoI and BamHI engineered on the 5’ and 3’ ends respectively, to facilitate excision from pJ201 (DNA 2.0’s cloning vector) and ligation into pET28 for expression. Insertion into pET28 at the XhoI site results in addition of a 6-histidine tag at the N-terminus to allow later purification of the expressed protein by nickel chromatography. In addition, the vector encodes a thrombin site for removal of the His tag, if desired, as well as a T7-derived epitope tag for detection of the recombinant protein. DNA was prepared in the NEB10B cells and protein was expressed in Rosetta2 cells. Note that the leader sequence for the K8.1 protein was maintained in the construct to allow for modification of the construct for eukaryotic cell expression if desired. These amino acid sequences are highly specific for bacteriophage T7 and HHV8, respectively. Potential cross-reactivity with antibodies in some HHV8-negative individuals is controlled by use of non-related recombinant proteins expressed in the same vector and purified in the same manner.

Bacteria transformed with each expression construct were grown at 37C and induced for expression by the addition of 5 mM IPTG when the cultures reached approximately 1 OD_550_. Expression was allowed to continue for 4 hours, at which time the bacteria were pelleted, then re-suspended in 10 mM Tris-HCl, pH 8 and frozen at -80C.

Cells were typically thawed the next day for protein purification and the solution was made 10% with NP40, then homogenized using a Waring^®^ blender for 30 seconds. The homogenate was then centrifuged at 8,000 x g for 1 hr. Supernatants were then decanted, filtered and subjected to nickel chromatography using chelating sepharose (GE Healthcare) saturated with NiS0_4_.

Sequences employed for each construct:

LANA:

MGSS**HHHHHH**SSGLVPRGSHMASMTGGQQMGRGSMEVEEQEEQELEEVEEQEEQELEEVEEQEQQGVEQQEQETVEEPIILHGSSSEDEMEVDYPVVSTHEQIASSPPGDNTPDDDPQPGPSREYRYVLRTSPPHRPGVRMRRVPVTHPKKPHPRYQQPPVPYRQIDDCPAKARPQHIFYRRFLGKDGRRDPKCQWKFAVIFWGNDPYGLKKLSQAFQFGGVKAGPVSCLPHPGPDQSPITYCVYVYCQNKDTSKKVQMARLAWEASHPLAGNLQSSIVKFKKPLPLTQPGENQGPGDSPQEMT*

K8.1:

MGSS**HHHHHH**SSGLVPRGSHMASMTGGQQMGRGSMSSTQIRTEIPVALLILCLCLVACHANCPTYRSHLGFWQEGWSGQVYQDWLGRMNCSYENMTALEAVSLNGTRLAAGSPSSEYPNVSVSVEDTSASGSGEDAIDESGSGEEERPVTSHVTFMTQSVQATTELTDALISAFSGSYSSGEPSRTTRIRVSPVAENGRNSGASNRVPFSATTTTTRGRDAHYNAEIRTHLYILWAVGLLLGLVLILYLCVPRCRRKKPYIV*

The HIV gp41 protein was a commercially sourced recombinant protein expressed in E. coli and purified by SDS-PAGE.

### Assay cartridge and instrument

The system consists of disposable cartridges and a simple reader instrument, based on planar waveguide imaging technology. The cartridge incorporates a microarray of recombinant antigens and antibody controls in a fluidic channel, providing multiple parallel fluorescence immunoassay results for a single sample[28]. A protein microarray was printed to the planar waveguide and subsequently assembled into an injection-molded, disposable cartridge as previously described[28]. The HIV / KSHV antibody detection cartridge comprised an array of three antigens: HIV-1 gp41 and KSHV antigens LANA and K8.1. The array also included controls with anti-human IgG (sample control) and printed human IgG (detect reagent control).

#### Assay reagents

Other reagents include purified human IgG (Sigma, St. Louis, MO), goat anti-human IgG (Thermo Scientific, Rockford, IL), and goat anti-human IgG conjugated with fluorescent dye (DyLight649; KPL, Inc.). Assay reagents include bovine serum albumin (BSA; Sigma Life Science, St. Louis, MO), phosphate-buffered saline (PBS), blocker casein in PBS, and Tween 20 (Thermo Scientific, Rockford, IL).

#### MBio system assay procedure

Samples were processed in cartridges on the benchtop at ambient temperature (20 to 25°C). Since the assay is independent of the reader instrument, sample cartridges were batch processed. A 6-μl aliquot of plasma was diluted in 194μl of diluent (PBS, 0.5% casein, 0.05% Tween 20). Of this diluted sample mixture, 175μl was loaded into the cartridge by transfer pipet, followed by a 15-minute incubation. One hundred seventy-five microliters of wash buffer (PBS, 0.1% Tween 20) was then added to the cartridge input port and allowed to flow through the cartridge for 3 minutes, followed by the addition of 175μl of dye-conjugated anti-human IgG in diluent (PBS, 1mg/ml BSA, and 0.05% Tween 20) and incubated for 10 minutes. Total assay time is approximately 30 minutes and a single operator can run 20 to 25 samples in parallel (batch mode). By comparison, time-to-result for the ELISA 96-well plate was approximately 2 hours with multiple manual interventions.

### Data Analysis

Antibody reactivity on the MBio System is based on a semi-quantitative analysis of signal / cutoff (S/CO). Cutoff values were determined for each viral antigen independently using the collection of commercially sourced negative sera described above. To establish cutoffs for the two KSHV antigens, mean and standard deviations for signal intensity were calculated for each antigen. Three of the 93 negative samples gave antibody-antigen reactivities that were greater than 3 standard deviations from the mean for the 93 sample set. These 3 samples were excluded, and the KSHV antigen cutoffs were re-calculated as the mean plus three standard deviations using the remaining 90 samples. By choosing to exclude 3 “high signal negatives” in the cutoff calculation, and by using distribution of 3 standard deviations instead of 5, we are biasing the assay for sensitivity at the potential cost of losing specificity. Cutoffs for the HIV gp41 feature were calculated using all 93 negative samples and calculating mean plus 5 standard deviations. The 5 standard deviation approach ensures the HIV Ab assay meets the specificity performance of widely used assays.

For the clinical HIV/KSHV samples, signal intensity on each antigen feature in the array was measured in the MBio System and Signal/Cutoff was calculated. S/CO > 1.05 is reported as positive; S/CO < 0.95 is reported as negative. Results with 1.05 ≥ S/CO ≥ 0.95 are reported as indeterminate. Microsoft Excel and GraphPad Prism software were used for statistical analysis. Mann-Whitney U tests were used to compare the MBio S/CO values among groups.

For the clinical analysis, simple linear regression was used to examine for potential univariate associations between the MBio S/CO of positive samples and corresponding patient characteristics, including KSHV PBMC and plasma viral load, CD4 count, HIV RNA viral load, gender, age, and KS stage (T1 and T0). Multiple linear regression models were used for multivariate results to adjust for potential confounders. Statistical analyses were performed using SAS^®^ version 9.2, assuming a two-sided significance level of 0.05.

## Results

### Clinical Samples

A total of 164 human plasma samples obtained from participants in studies of KS conducted at the University of Zimbabwe College of Health Sciences were used in this study (Table 1). Demographic characteristics of the patients from whom the samples were obtained included a median age of 37 years; 65% were from males; KS tumor stage was T0 in 23% of participants and T1, or more advanced disease, in 77%. Of the samples with contemporaneous immunologic and virologic parameters measured, the median CD4+ T-cell count was 143 cells/μl and the median plasma HIV-1 RNA level was 4.59 log copies/mL. The median KSHV plasma DNA level was 1.78 log copies/mL and the median PBMC KSHV DNA level was 3.24 log copies/10^5^ cells.

**Table 1:** Characteristics of Study Participants.

Median age, (years) (range) n = 163	37 (20–71)
Gender, (%) n = 163	
Male	106 (65.0)
Female	57 (35.0)
KS tumor stage, (%) n = 162	
T0 (localized disease)	38 (23)
T1 (more advanced disease)	124 (77)
Median CD4 T cell count, cells/μl, (range) n = 157	143 (0–906)
Median Plasma HIV-1 RNA level, log copies/mL (range) n = 49	4.59 (2.60–5.54)
Median Plasma KSHV DNA level, log copies/mL, (range) n = 163	1.78 (0.99–4.55)
Median PBMC KSHV DNA level, log copies/10^5^ cells, (range) n = 148	3.24 (1.00–7.52)

### KSHV ELISA Results

KSHV antibody reactivity by ELISA was positive in 158 of 164 biopsy-proven KS-positive samples (96.3% sensitivity, 95% CI = 93.5% to 99.2%). One sample was reported as equivocal / borderline. None of the 93 control sera showed KSHV antibody reactivity (100% specificity).

### MBio Array System Results

#### KSHV antibody reactivity

**Fig 1:**
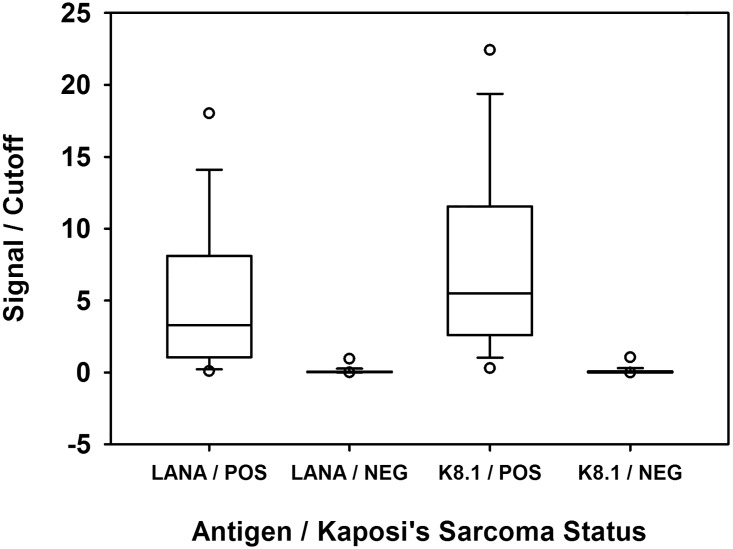
Signal / Cutoff values for the KSHV antigens LANA and K8.1 in the MBio Array cartridge. “POS” indicates the Kaposi’s Sarcoma biopsy-positive samples. “NEG” indicates negative control sera as determined by KSHV ELISA. The box and whisker plots show the median, 10^th^, 25^th^, 75^th^ and 90^th^ percentiles. The circles show location of the 5^th^ and 95^th^ percentiles.

**Fig 2:**
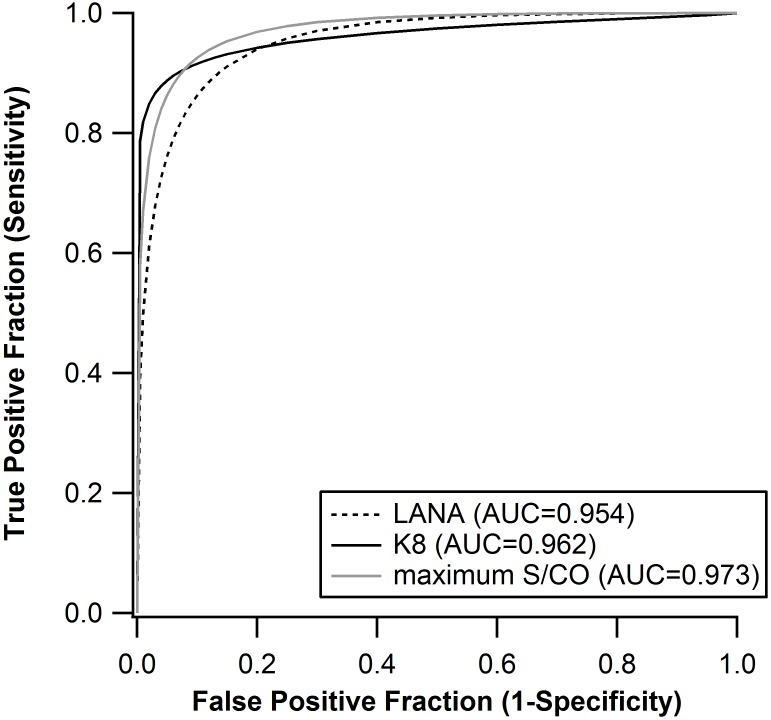
ROC curve for MBio assay, including individual antigens, LANA and K8.1 and maximum S/CO.

KSHV antibody reactivity (S/CO) results are presented in Fig 1. Receiver-Operator Characteristic (ROC) curves were generated for each individual antigen and for the maximum signal/cutoff value and are provided in Fig 2. The median S/CO for the LANA antigen in the KS-positive samples was 3.17, which was > 100-fold higher than the median S/CO of the controls with a median S/CO of 0.03 (Mann-Whitney U test 741.0, P < 0.0001). The median S/CO for the K8.1 antigen in the KS-positive samples was 7.26, which was > 200-fold higher than the median S/CO of the negative controls with a median S/CO of 0.03 (Mann-Whitney U test 555.0, P < 0.0001).

Considered independently, the MBio K8.1 assay reported antibody reactivity for 152 of 164 KS-positive samples (sensitivity = 92.7%; 95% CI = 88.7% to 96.7%) and called 87 of 93 control samples as negative (specificity 93.5%; 95% CI = 88.6% to 98.5%). The LANA assay reported 3 indeterminate results (1.17% indeterminate rate) and reported antibody reactivity for 122 of 161 KS-positive samples (sensitivity = 75.8%; 95% CI = 69.2% to 82.4%) and called 89 of 93 control samples as negative (specificity 95.7%; 95% CI = 91.6% to 99.8%). The LANA has significantly lower sensitivity than K8.1. We note here that the specificity analysis below is non-ideal in that it is based on a self-referential dataset, i.e., the control samples used to establish cutoffs are the same samples used in the specificity calculations.

Relatively low correlation of S/CO is observed between K8.1 and LANA. For the collection of 164 biopsy-proven KS samples, the Pearson Product-Moment Correlation Coefficient for K8.1 and LANA S/CO values is 0.20. One could thus expect that a combination of two antigens could improve overall assay sensitivity. In fact, three KS-positive samples that were negative on the K8.1 assay gave positive reactivity on the LANA assay (one K8.1 negative was indeterminate on LANA). Given this information, a two-antigen KSHV algorithm was applied in which a sample is considered positive if either (or both) of the K8.1 and LANA antigens show reactivity. With this dual antigen algorithm approach, antibody reactivity was reported on 155 of 163 KS-positive samples (sensitivity = 95.1%; 95% CI = 91.0% to 98.0%). One sample from a participant with active KS was reported as KSHV indeterminate using the dual antigen algorithm (0.4% indeterminate rate). The dual antigen algorithm reported 85 of 93 control samples as negative. The dual antigen assay produced two addition false positive results relative to the K8.1 assay (8 versus 6), indicating a slight decrease in specificity. Of the false positive control samples, 4 were positive on the K8.1 assay, 2 on the LANA, and 2 on both assays. All were negative on the ELISA. Defining accuracy as (true positives + true negatives)/total, results are 82.1% for LANA, 92.7% for K8.1, and 93.4% for the dual antigen approach. We note that the K8.1 alone and the dual antigen algorithm are statistically equivalent for this dataset.

Comparing specific samples between MBio and ELISA, 3 of 8 MBio false negatives (no Ab reactivity in KS-positive samples) were also negative by ELISA. The ELISA thus has slightly better sensitivity in the KS-positive sample collection than the MBio assay (96.3% versus 95.1%, although both are within 95% CI). Of the remaining five samples, the OD/cutoff ratio on the ELISA platform ranged from 1.10 to 8.99, with a median OD/cutoff of 3.47.

For samples from patients with KS that were identified as KSHV antibody positive, both antigens demonstrated robust antibody titers. The median S/CO for K8.1 was 8.16, which was significantly higher than the median S/CO for LANA at 5.33 (Mann-Whitney U test 6753, p 0.0001). The LANA antigen identified 122 positive samples (74.4%). By including the K8.1 antigen on the array 152 positive samples were identified, increasing the sensitivity of the assay to 92.7%.

#### HIV-1 antibody reactivity

The MBio Array System simultaneously assesses HIV-1 and KSHV antibody reactivity for each sample. Of the 164 HIV-seropositive samples from KS patients, all 164 were reported as HIV-1 Antibody reactive on the MBio assay. A total of 66 HIV-seronegative samples were tested with the HIV assay. One of the 66 was indeterminate, and 65 of 66 were non-reactive.

### Multivariate Analysis

Clinical data were available for 163 samples with both MBio S/CO and ELISA O.D. level results. The KS positive samples were evaluated for potential association with KSHV PBMC and plasma viral loads, CD4 count, HIV RNA viral load, gender, age and KS stage (T1 and T0). In the univariate analysis, a higher MBio S/CO level was significantly associated with having a T1 KS stage (p = 0.02). Male gender and having a higher KSHV plasma viral load also showed association, although not significant (both p<0.09). In multivariate modeling, after adjusting for all clinical variables, only being male (p = 0.04) remained significant. The association with T1 KS stage was no longer significant and the clinical variables explained only 5% of the variability in the final multivariate model (Table 2).

**Table 2:** Multivariable-adjusted associations between clinical factors and MBio S/CO and ELISA O.D. levels.

	MBio S/CO level *		ELISA O.D. level **	
	Coefficient (SE)	p-value	Coefficient (SE)	p-value
Age (years)	-0.01 (0.11)	0.93	0.02 (0.04)	0.61
Gender (male)	-3.93 (2.01)	0.04	-2.30 (0.79)	0.004
KS Stage (T1)	2.64 (2.51)	0.29	2.20 (0.99)	0.03
CD4 T cell count, cells/μl,	0.00 (0.01)	0.65	0.00 (0.00)	0.21
Plasma KSHV DNA level, log copies/mL	1.21 (1.21)	0.32	-0.25 (0.48)	0.60
PBMC KSHV DNA level, log copies/10^5^ cells	0.24 (0.66)	0.72	-0.53 (0.26)	0.04

* R^2^ = 0.05;

** R^2^ = 0.12

In univariate analysis, a higher ELISA O.D. level was significantly associated with being male (p = 0.03), having T1 KS stage (p = 0.01) and higher HIV RNA viral load (p = 0.04). In multivariate modeling, after adjusting for all clinical variables, being male (p = 0.004), T1 KS stage (p = 0.03), and KSHV PBMC viral load (p = 0.04) were significant (Table 2). However, the clinical variables explained only 12% of the variability in the multivariate model. HIV RNA viral load was not included in the since results were available for less than a third of the cohort.

## Discussion

Knowledge of co-infection status with several critical co-pathogens in those with HIV-1 infection could be of substantial value in devising optimal management strategies for those with one or more of these co-infections, including strategies to prevent malignancies associated with them. Based on the evidence that effective antiretroviral therapy decreases the incidence of KS, it is hypothesized that early identification and antiretroviral treatment of HIV-infected persons with KSHV would substantially reduce KS-associated morbidity and mortality[8, 42–45]. Despite improvements in detection, serologic tests are rarely used clinically to identify those who have sub-clinical infection and are thus at risk for development of KS, due to cost and general lack of access to central laboratories in the places where the virus is most prevalent. A readily accessible and affordable method for the identification of individuals with KSHV and other co-infections would allow clinicians to factor this information into clinical decision-making, potentially starting ART at higher CD4 cell counts in areas where this is not yet routine despite updated guidelines for universal treatment, or increasing KS surveillance for earlier detection as strategies to reduce morbidity and mortality.

In this initial study, the MBio serodiagnostic assay showed 95.1% sensitivity in detecting antibody in plasma from patients with KS. Robust signals were detected with both the K8.1 and LANA antigens, but the S/CO was higher for K8.1 than for LANA. In addition, more samples demonstrated reactivity to the lytic K8.1 antigen, which has also been shown in previous studies, and is expected due to replicating virus in the setting of active KS disease[36, 37, 46]. The results are also supported by our finding that the S/CO was higher in individuals with more advanced T1 KS stage, though this did not remain significant in the multivariate analysis. We also demonstrated that parameters associated with advanced HIV disease were not associated with KSHV S/CO values as we might have expected.

One limitation of this study is that only samples from patients with biopsy-proven KS were used to assess performance of the assay. Overestimation of performance is a concern, due to higher antibody titers in KS patients compared to those with asymptomatic KSHV infection[24, 47–54]. Future studies utilizing additional cohorts including those with asymptomatic KSHV infection are necessary to confirm the accuracy of this approach.

Another limitation is the use of only 2 antigens on the array. Prior studies demonstrated the additional benefit of using v-cyclin, as well as other KSHV proteins[9, 23, 25, 27, 55, 56], but v-cyclin was not included in this analysis because of difficulties in printing it onto the array. Three of the eight KS-positive samples that were classified as negative by the MBio assay were also negative on the ELISA platform. Of the remaining 5 samples, the ELISA signals varied, ranging from 1.10 to 8.99. This suggests that the higher sensitivity of the ELISA platform might be attributable to additional antigens present in a whole-viral lysate that were not represented in the two-antigen MBio array. Thus it might be possible to increase the sensitivity of the assay by expanding the antigens included in the array. Because the primary goal of this initial study was feasibility, a truncated LANA protein was expressed to improve yield, and a bacterial expression system was selected for ease and cost. Sensitivity of the assay may also be improved by using the full length LANA protein and use of insect or mammalian cells for protein expression, and will be considered in future studies.

The MBio system can simultaneously measure up to 30 markers, so addition of other immunological targets to improve the sensitivity and specificity can be readily implemented. Another major advantage of this multiplexed system is the ability to simultaneously assess for the presence of other co-infections. This omits the need for multiple testing platforms to screen for co-infections, which both simplifies the testing process and conserves sample volumes. With further optimization, this system can potentially be implemented into the point of care, such that knowledge of co-infections may be used for clinical decision-making, and can also be used in blood donor screening and in epidemiologic studies evaluating HIV and co-infections in various populations.

In conclusion, these preliminary results indicate that the MBio system can detect antibody titers to KSHV antigens with good sensitivity and specificity approaching that of a commercially available ELISA. Further studies will be performed to evaluate the benefit of additional antigens on the array and to investigate antibody profiles in additional cohorts, including PEL, MCD, and especially asymptomatic infection. Once the optimal antigens are selected, more formal validation studies will be performed, specifically to evaluate both accuracy and reproducibility of the improved assay. Additional studies are ongoing to optimize the assay for screening of HIV-1, KSHV and other important co-infections for use in clinical care in resource-limited settings.

## Supporting Information

S1 File KSHV Manuscript Data File. (XLSX)
